# Probing Sub-atomistic Free-Volume Imperfections in Dry-Milled Nanoarsenicals with PAL Spectroscopy

**DOI:** 10.1186/s11671-016-1228-9

**Published:** 2016-01-12

**Authors:** Oleh Shpotyuk, Adam Ingram, Zdenka Bujňáková, Peter Baláž, Yaroslav Shpotyuk

**Affiliations:** Vlokh Institute of Physical Optics, 23, Dragomanov str., 79005 Lviv, Ukraine; Jan Dlugosz University, al. Armii Krajowej, 13/15, 42201 Czestochowa, Poland; Opole University of Technology, 75, Ozimska str., 45370 Opole, Poland; Institute of Geotechnics of the Slovak Academy of Sciences, 45, Watsonova str., 04001 Košice, Slovakia; Ivan Franko National University of Lviv, 107, Tarnavskogo str., 79017 Lviv, Ukraine; Centre for Innovation and Transfer of Natural Sciences and Engineering Knowledge, University of Rzeszow, 1, Pigonia str., 35-959 Rzeszow, Poland

**Keywords:** Nanomaterial, Free-volume void, Positron annihilation, Positron trapping

## Abstract

Structural transformations caused by coarse-grained powdering and fine-grained mechanochemical milling in a dry mode were probed in high-temperature modification of tetra-arsenic tetra-sulfide known as β-As_4_S_4_. In respect to X-ray diffraction analysis, the characteristic sizes of β-As_4_S_4_ crystallites in these coarse- and fine-grained powdered pellets were 90 and 40 nm, respectively. Positron annihilation lifetime spectroscopy was employed to characterize transformations occurred in free-volume structure of these nanoarsenicals. Experimentally measured positron lifetime spectra were parameterized in respect to three- or two-term fitting procedures and respectively compared with those accumulated for single crystalline realgar α-As_4_S_4_ polymorph. The effect of coarse-grained powdering was found to result in generation of large amount of positron and positronium Ps trapping sites inside arsenicals in addition to existing ones. In fine-grained powdered β-As_4_S_4_ pellets, the positron trapping sites with characteristic free volumes close to bi- and tri-atomic vacancies were evidently dominated. These defects were supposed to originate from grain boundary regions and interfacial free volumes near aggregated β-As_4_S_4_ crystallites. Thus, the cumulative production of different positron traps with lifetimes close to defect-related lifetimes in realgar α-As_4_S_4_ polymorph was detected in fine-grained milled samples.

## Background

Positron annihilation lifetime (PAL) spectroscopy is high-informative tool in studying sub-atomistic free-volume imperfections in solids affected by different nanostructurization routes [1–5]. With complementary mathematical algorithms allowing correct parameterization of mixed positron-electron annihilation paths in structurally complicated substances, this method (the positronics [6]) can be successfully motivated as a nanoscale alternative for conventional micro-meso-scale porosimetry exemplified by such well-approbated techniques as gas (nitrogen) sorption, mercury intrusion, and small-angle X-ray scattering [7–9]. Undoubtedly, further progress in this field relies on stretching possibilities for positronics to be applied for a great diversity of known nanomaterials. In this work, we track this for principally different nanostructurized objects, these being coarse- and fine-grained powdered pellets of the same high-temperature polymorph of tetra-arsenic tetra-sulfide β-As_4_S_4_ extensively studied recently in view of promising anticancer functionality [10–14].

## Methods

The preliminary melt-quenched As_50_S_50_ alloy was used for further powdering, the known high-temperature modification of tetra-arsenic tetra-sulfide β-As_4_S_4_ being dominated in this precursor.

Firstly, the small bulk pieces of this arsenical were subjected to coarse-grained powdering and sieved under 200 μm. Then, the obtained powder was compressed by compacting inside a stainless steel die under a pressure of ~0.7 GPa to produce pellets having near 6 mm in a diameter and 1 mm in a thickness. This batch of pellets composed of coarse-grained powdered (CGP) β-As_4_S_4_ polymorph was conditionally termed as β-CGP.

Other part of the prepared β-CGP was subjected to high-energy milling, which is a very effective mode of treatment of solids [15, 16]. Dry mode of treatment using planetary ball mill Pulverisette 6 (Fritsch, Germany) under a protective argon atmosphere has been applied. The preliminary powdered substance (3 g) was put into tungsten carbide WC chamber with 50 milling balls (each of *d* = 10 mm in a diameter) made of the same WC material. The ball-to-powder weigh ratio was 120:1, and total duration of milling performed under rotation speed of 500 rpm was 60 min. After this milling route, the fine-grained powder (FGP) marked as β-FGP was pelletized under the same conditions as described above.

The crystallographical specificity of the pellets was identified with X-ray powder diffraction (XRPD), the experimental data being collected in a transmission mode using STOE STADI P diffractometer (STOE & Cie GmbH, Darmstadt, Germany) with Cu Kα_1_-radiation as was described in more details elsewhere [14]. The crystal structures of the phases were refined by the Rietveld method with the FullProf.2 k (v.5.40) program [17]. The microstructure properties of the revealed phases (average apparent crystallite size *D*, e.g., size of coherently diffracting domains, average maximum strain *S*) were defined during the Rietveld refinement procedure by isotropic line broadening analysis implemented in this program [18].

The PAL measurements were performed for pelletized β-CGP and β-FGP samples using fast-fast coincidence system ORTEC of 230 ps resolution (the full width at half maximum) based on two Photonis XP2020/Q photomultiplier tubes coupled to BaF_2_ scintillator 25.4A10/2M-Q-BaF-X-N detectors (Scionix, Bunnik, Holland) and ORTEC^®^ electronics (ORTEC, Oak Ridge, TN, USA). The radioactive ^22^Na isotope of low activity (~50 kBq) wrapped by the Kapton^®^ foil (DuPont™, Circleville, OH, USA) and sealed was used as positron source sandwiched between two identical pellets. The normal-measurement statistics arranged for near 1 M elemental positron annihilation events collected at high-stabilized temperature of 22 °C and relative humidity of 35 % was employed to ensure reliable PAL measurements. The channel width of 6.15 ps allows a total number of available channels to be 8000. Three separate measurements ensure a good reproducibility of this research, the source contribution being evidenced at the level of 15 % allowing practically full compensation of input from positrons annihilated in the Kapton^®^ foil with a lifetime of 0.372 ns.

The obtained PAL data were fitted by two (×2-decomposition) or three (×3-decomposition) single exponents under unity-normalized intensities using LT 9.0 program [19], the accuracies in lifetimes *τ*_*i*_ and intensities *I*_*i*_ being not worse ±0.005 ns and 0.5 %, respectively. Positron trapping formalism developed in terms of known two-state model with only one kind of defects [1–3, 20, 21] was utilized to parameterize mean *τ*_*av*_ and defect-free bulk *τ*_*b*_ lifetimes, as well as trapping rate in defects *κ*_*d*_, which was determined under above measurement conditions with ±0.01 ns^−1^ accuracy. In addition, the difference between defect-related *τ*_*d*_ = *τ*_*2*_ and defect-free positron lifetimes (*τ*_*2*_–*τ*_*b*_) was taken as a signature of size of extended positron traps in terms of equivalent number of vacancies, whereas *τ*_*2*_/*τ*_***b***_ ratio was ascribed to the nature of these defects [1]. In loosely packed media like polymers or molecular substances, the positrons can also annihilate from bound positron-electron (positronium (Ps)) states through pick-up an electron from an environment [1, 2, 20, 22]. In respect to known Tao-Eldrup formalism [1, 2], the localized Ps gives an indication on corresponding free-volume void radius *R* in terms of long-lived *τ*_*3*_ lifetime.

## Results and Discussion

**Fig. 1:**
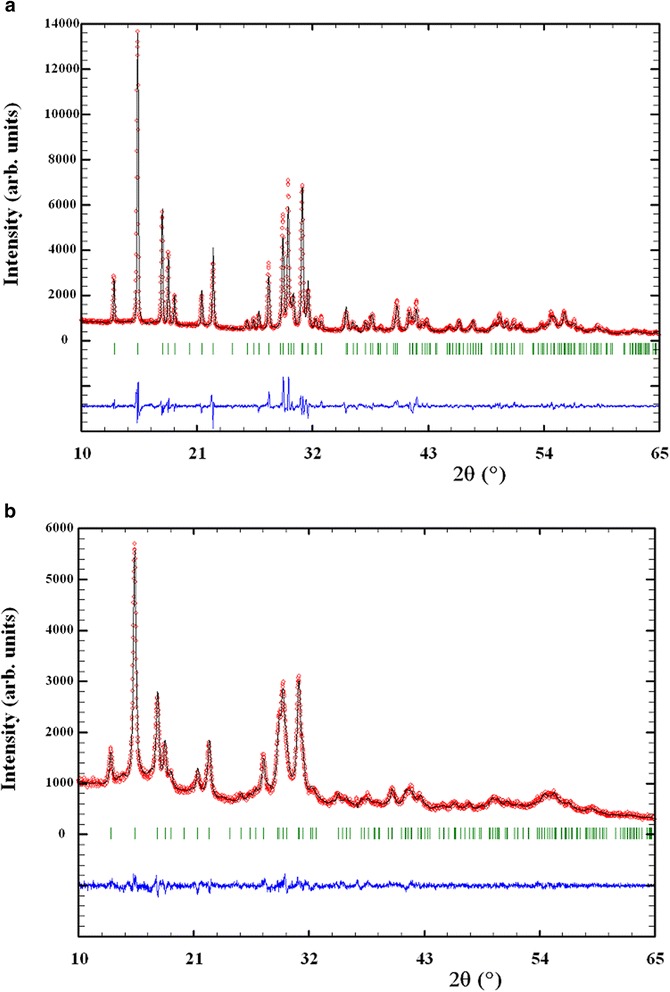
Observed (*circles*) and calculated (*solid line through circles*) XRPD profiles for β-CGP (**a**) and β-FGP (**b**) pellets given with calculated Bragg positions (*vertical ticks*) for β-realgar As_4_S_4_; the difference curve is given at the bottom (*solid line*)

The XRPD patterns of β-CGP and β-FGP pellets are shown in Fig. 1, top and bottom, respectively. The same β-As_4_S_4_ phase of C2/c space group was obviously dominated in both pellets (β-CGP and β-FGP), giving two different sets of crystallographic lattice parameters:

- *a* = 9.9200(2), *b* = 9.3946(2), *c* = 8.9505(2) Å, and *β* = 101.968(2)° for β-CGP and
- *a* = 9.9047(5), *b* = 9.4173(5), *c* = 9.0133(5) Å, and *β* = 101.246(4)° for β-FGP.

The lattice parameters of synthetic β-As_4_S_4_ are known to differ essentially in dependence on the preparation conditions [23]. The above lattice parameters are close to those observed in one of synthetic β-As_4_S_4_ prepared by Roland [24]. It is worth mentioning that effect of nanomilling in its crystallographic appearance occurs to be identical with light-induced alteration of β-As_4_S_4_ in pararealgar [23]. In both cases, the lattice parameters show similar tendencies revealing decrease in {*a, β*} and increase in {*b, c*} values. We are far from a mind on full identity between nanostructural transformations caused by light exposure and mechanochemical milling, but this result undoubtedly testifies that initial stages of both processes, connected with introducing structural disordering in a crystalline network, are indeed very similar.

The average apparent crystallite sizes *D* estimated for main reflexes of β-As_4_S_4_ phase (Fig. 1) approach 90.5 and 40.2 nm, while average maximum strains *S* achieve 0.0030 and 0.0063 for β-CGP and β-FGP, respectively. Thus, the nanomilling does not change preferential crystalline state of this arsenical but produce an obvious effect consistent with essential decrease in a size of β-As_4_S_4_ crystallites with an accompanied increase in inner strains.

Such changes in atomistic structure caused by high-energy mechanochemical milling are apparently concomitant with generation of structural defects acting as eventual positron and Ps traps in a bulk source material [4]. So, in the analysis of PAL data reflecting these possible positron-Ps traps, it is reasonably to refer the previous PAL study for monolith prototype of these pellets, such as mineral realgar α-As_4_S_4_, the known room-temperature polymorph of arsenic sulfide As_4_S_4_ [25]. Both these crystallographic modifications (α-As_4_S_4_ and β-As_4_S_4_) are substantially identical from point of their main structural fragments (the elementary cage-type As_4_S_4_ molecules possessing *D*_2*d*_ symmetry); they differ only by molecular packing leading to two different monoclinic lattices [26]. So, both polymorphs occur to be very similar in terms of their *volume-per-atom* determination. Indeed, the calculated crystallographic densities are 3.56 and 3.52 g ⋅ cm^−3^ for α-As_4_S_4_ and β-As_4_S_4_, respectively [27], giving nearly the same free volume averaged per one atom (~25.0 Å^3^).

As was shown previously [25], positron annihilation in the medium filled with cage-like As_4_S_4_ molecules (as in α-As_4_S_4_) is defined by extended free-volume positron trapping centers in the form of outer overlapped spaces attached to neighboring S atoms forming rectangular bypass line around As_4_S_4_ molecule. Such spaces possess effective negative charges (in view of electronegativity of S atoms in heteronuclear As–S bond), which makes them preferential traps due to attractive potential for positrons [3, 28]. Similar free-volume configurations are supposed to be characteristic for many other arsenic sulfide compounds such as crystalline pararealgar As_4_S_4_, orpiment As_2_S_3_, or even near-stoichiometric glassy As–S, ensuring close similarity in their defect-related lifetimes in 0.34–0.37 ns domain [3, 25, 28–30]. An alternative channel of positron annihilation is expected in realgar α-As_4_S_4_ for Ps decaying in free volumes derivative from crystallographic-specific packing of cage-like As_4_S_4_ molecules. However, overall Ps yield in realgar is rather small (2–3 %) [25]; thus, the detected PAL spectra are dominated by preferential positron trapping.

The experimental PAL spectra of bulk realgar α-As_4_S_4_ can be well fitted with three single exponents evolving inputs from positron and Ps trapping, the corresponding trapping modes for ×3-deconvolution procedure being gathered in Table 1 [25]. This crystal demonstrates high enough defect-related lifetime *τ*_*2*_ = 0.346 ns, *τ*_*2*_*/τ*_*b*_ ratio approaching 1.54 and (*τ*_*2*_*-τ*_*b*_) difference near 0.12 ns, which can be evidently attributed to relatively large ~80 Å^3^ free-volume voids (compared to bi- and tri-atomic vacancies) as it follows from known analytical correlations for this type of chemical environment [30–32]. The calculated defect-free bulk lifetime *τ*_*b*_ = 0.224 ns correlates well with this parameter in similar crystalline arsenicals, such as orpiment As_2_S_3_ (*τ*_*b*_ = 0.242 ns) [3], but is substantially smaller than *τ*_*b*_ ≅ 0.28–0.29 ns in glassy As_2_S_3_ with higher content of free volumes [3, 28]. However, in contrast to these compounds, the channel of o-Ps decaying with *τ*_*3*_ ≅ 1.873 ns lifetime is more pronounced in mineral realgar α-As_4_S_4_, while the corresponding intensity is still no more 2.6 %. Unambiguous identification of Ps-decaying channel in the reconstructed PAL spectra under such low *I*_*3*_ is problematic, since this component can be admixed with uncontrolled contribution from a source [1, 2]. By inserting whole input from o-Ps decaying directly to the source, this task can be removed to other ×2-decomposition (Table 1), which results in definitely smallest defect-free bulk lifetime *τ*_*b*_ because of uncompensated input from p-Ps decaying in the first channel. This procedure leads to nearly the same *τ*_*b*_ ≈ 0.223 ns, which can be accepted as a lower limit of defect-free bulk positron lifetime in realgar α-As_4_S_4_. The maximal value of *τ*_*b*_ for α-As_4_S_4_ can be obtained by transferring to generalized ×2-decomposition (×2-gen. row in Table 1), where all trapping channels (originated from positron, o-Ps and p-Ps decaying) contribute to one defect-related component [4, 6].

**Table 1:** Fitting parameters and corresponding PAL trapping modes describing positron annihilation in bulk mineral α-As_4_S_4_ and powdered β-As_4_S_4_

Sample, fitting	Fitting parameters						PAL trapping modes			
	*τ*_*1*_	*I*_1_	*τ*_2_	*I*_2_	*τ*_3_	*I*_3_	*τ*_*b*_	*κ*_*d*_	*τ*_2_ *-τ*_*b*_	*τ*_2_ */τ*_*b*_
	ns	a.u.	ns	a.u.	ns	a.u.	ns	ns^−1^	ns	a.u.
α-As_4_S_4_, ×3	0.193	0.666	0.346	0.308	1.873	0.026	0.224	0.72	0.12	1.54
α-As_4_S_4_, ×2	0.193	0.685	0.339	0.315	–	–	0.223	0.70	0.12	1.52
α-As_4_S_4_, ×2-gen.	0.194	0.656	0.456	0.343	–	–	0.241	1.02	0.22	1.89
β-CGP, ×3	0.207	0.745	0.432	0.222	2.337	0.033	0.235	0.58	0.20	1.84
β-CGP, ×2	0.206	0.793	0.439	0.207	–	–	0.232	0.53	0.21	1.89
β-CGP, ×2-gen.	0.208	0.734	0.656	0.266	–	–	0.254	0.87	0.40	2.58
β-FGP, ×2	0.193	0.607	0.344	0.393	–	–	0.233	0.90	0.11	1.48

**Fig. 2:**
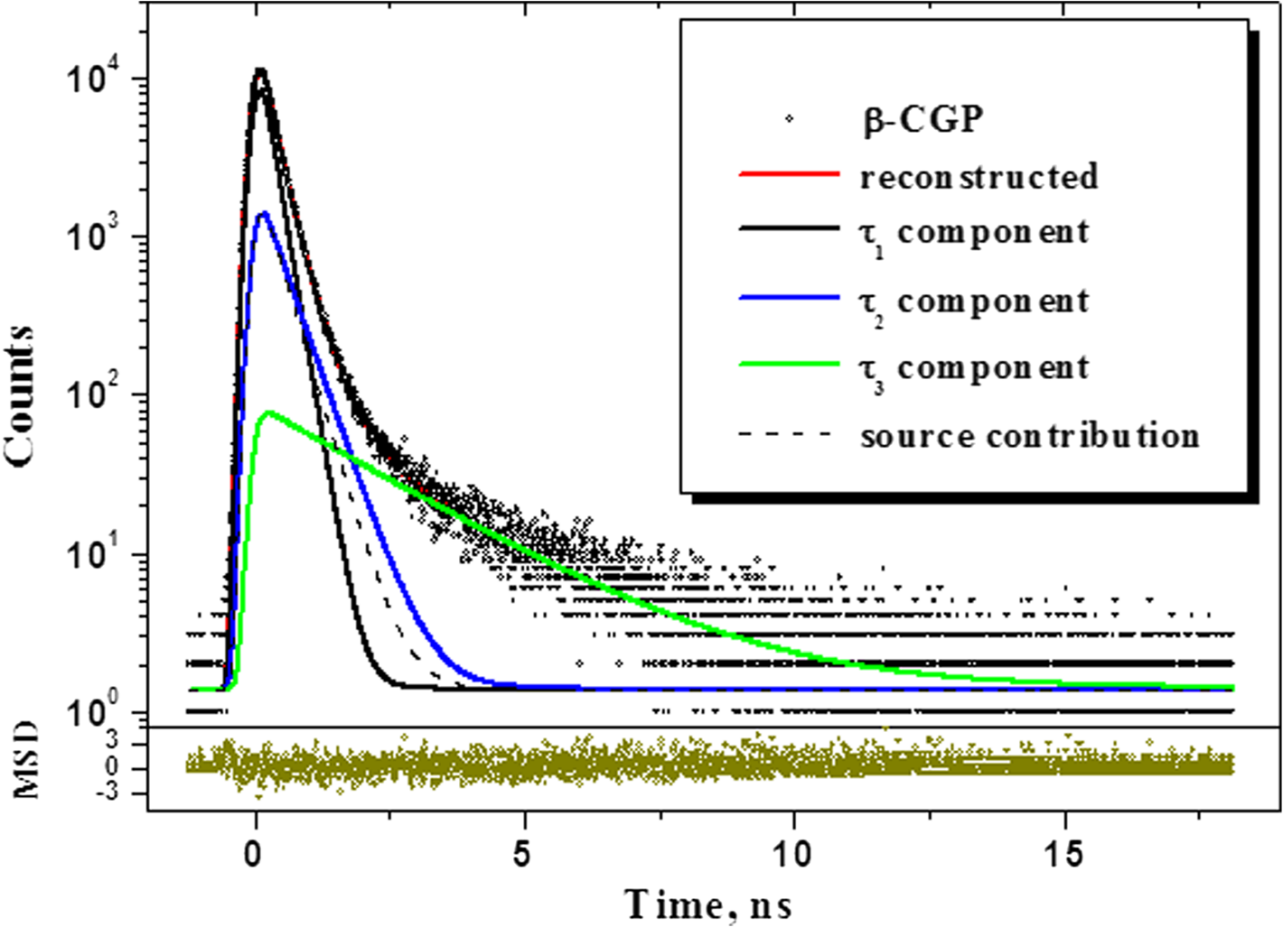
Raw PAL spectrum of β-CGP pellet reconstructed from ×3-fitting procedure at the general background of standard source contribution

**Fig. 3:**
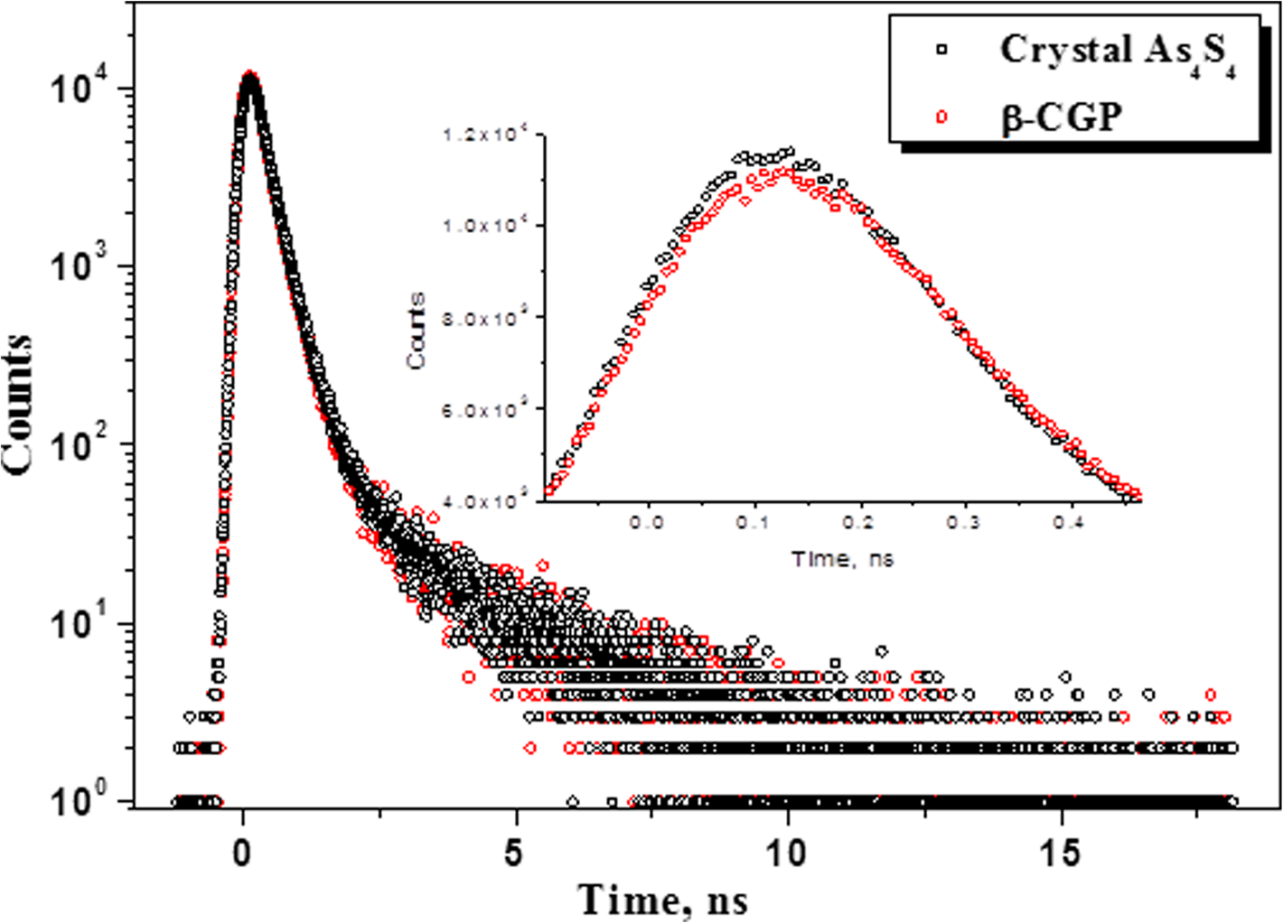
Comparison of raw PAL spectra for mineral realgar α-As_4_S_4_ [25] and pelletized β-CGP, the *inset* shows depressed peak intensity in β-CGP as compared to realgar (see text for details)

The raw PAL spectrum of β-CGP pellets reconstructed from ×3-fitting procedure is shown in Fig. 2, and corresponding trapping modes are given in Table 1. As compared to realgar α-As_4_S_4_, the positron annihilation essentially changes in this nanostructurized arsenical, especially in respect to positron trapping channel. For a more detailed analysis, the PAL spectra of both arsenicals (the mineral realgar α-As_4_S_4_ [25] and pelletized β-CGP) are compared on Fig. 3. It is obvious that the main changes occurred in β-CGP are related to depressed peak and increased slope in the histogram of annihilation counts. This tendency respectively inhibits positron trapping in β-CGP, as it follows from over 30 % reduction in *I*_*2*_ intensity, and nearly the same increase in defect-related *τ*_*2*_ lifetime. The channel of o-Ps decaying in β-CGP is also under significant modification, demonstrating slight increase in both *τ*_*3*_ lifetime and *I*_*3*_ intensity (Table 1). The calculated values of defect-free bulk lifetime *τ*_*b*_ = 0.235 ns is slightly enhanced as in realgar but still in a framework of above deviation for arsenicals 0.223 < *τ*_*b*_ < 0.241 ns. By inserting the o-Ps input directly to the source contribution, we obtained ×2-decomposition, where above positron trapping tendency was further enhanced (increase in *τ*_*2*_ and decrease in *I*_*2*_). It probably means that both positron and Ps trapping channels contribute to PAL spectra cumulatively, producing overall changes due to more stretched row of enlarged positron-Ps traps. Significant role of Ps decaying also follows from long-lived positron lifetime *τ*_*2*_ exceeding characteristic level of intrinsic vacuum Ps decaying (0.5 ns) [1, 2] obtained under generalized ×2-decomposition procedure (marked as ×2-gen. in Table 1). With reference to nanostructurization processes in similar substances [4, 21, 22], we can reasonably speculate that these trapping centers in the pelletized β-CGP can be identified as grain boundaries and intergranular voids originated directly from coarse powdering.

**Fig. 4:**
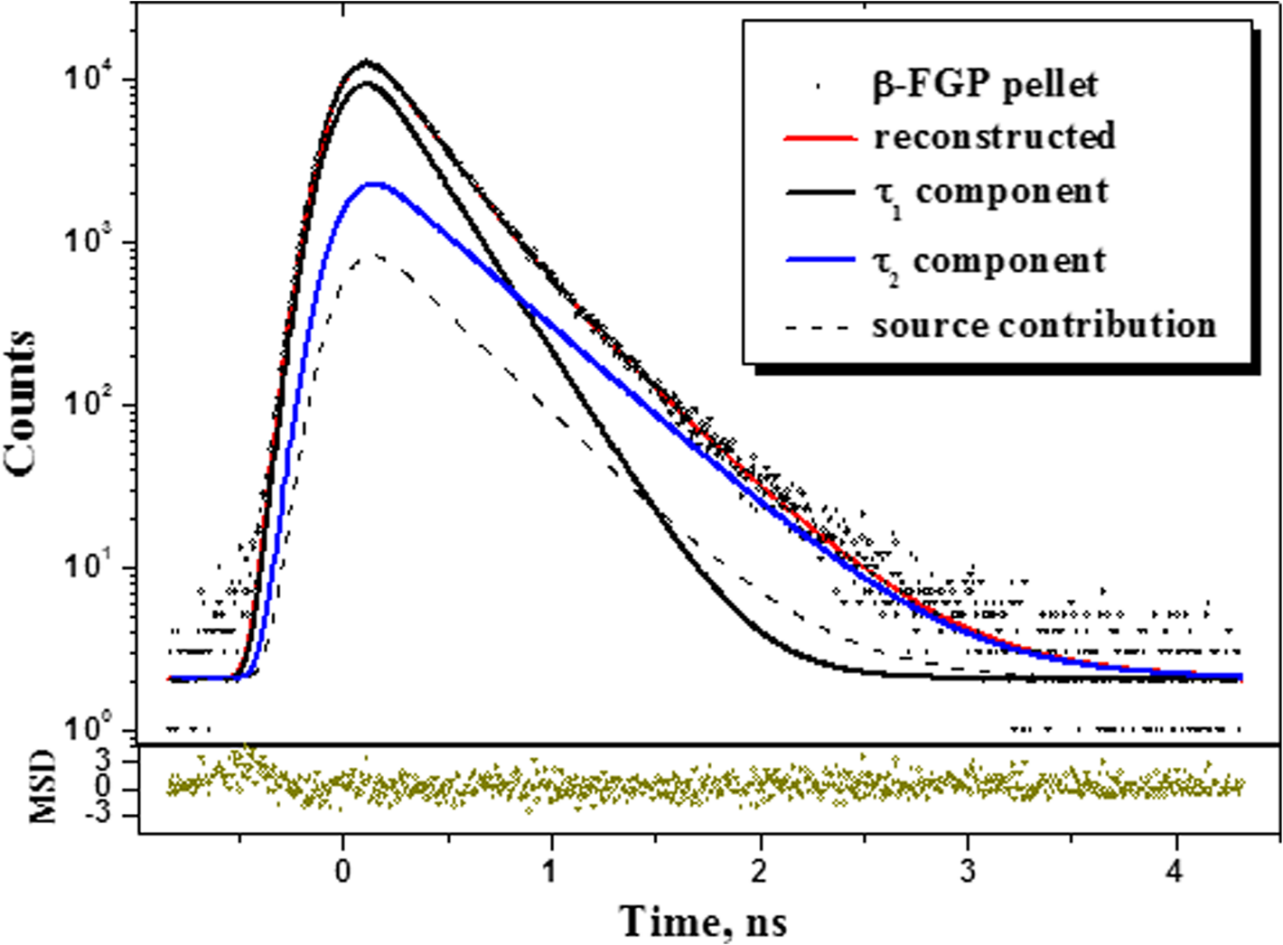
Raw PAL spectrum of β-FGP pellet reconstructed from ×2-fitting procedure at the general background of standard source contribution

With transition to mechanochemically milled arsenical (β-FGP), the void structure of pelletized β-CGP is subjected to more substantial modification. The raw PAL spectrum of β-FGP (see Fig. 4) can be satisfactorily reconstructed only from ×2-fitting procedure, the corresponding fitting parameters and trapping modes being gathered in Table 1. Such changes are concomitant, in the first hand, with disappearing of some Ps-related traps, which were more efficient in coarse-grained β-CGP pellets. Surprisingly, the bulk defect-free positron lifetime is not changed (*τ*_*b*_ = 0.233 ns), and both *τ*_*1*_ and *τ*_*2*_ lifetimes approach very close those in realgar α-As_4_S_4_ (so only component intensities *I*_*1*_ and *I*_*2*_ are subjects to more essential changes). Such behavior testifies that most rough positron-Ps trapping centers of β-CGP disappear under high-energy ball milling, so that the remaining ones contribute along with native realgar-type positron traps (both with nearly the same bulk positron lifetime *τ*_*b*_ = 0.233 ns) to cumulative positron trapping in β-FGP.

## Conclusions

Method of PAL spectroscopy employing conventional three- and two-term fitting was utilized to study free-volume structure of β-As_4_S_4_ arsenical subjected to coarse- and fine-grained powdering. The pelletized samples of coarse-grained powdered β-As_4_S_4_ demonstrate a great variety of possible positron and Ps trapping sites. Transition to fine-grained powdered β-As_4_S_4_ due to high-energy mechanochemical milling results in cumulative production of preferential positron traps with characteristic lifetimes close to defect-related lifetimes in crystalline realgar α-As_4_S_4_ polymorph. These positron traps were supposed to originate from grain boundaries and interfacial free volumes appeared near aggregated β-As_4_S_4_ crystallites.

## Abbreviations

CGP: coarse-grained powder
FGP: fine-grained powder
PAL: positron annihilation lifetime
XRPD: X-ray powder diffraction
